# Lisdexamfetamine Therapy in Paroxysmal Non‐kinesigenic Dyskinesia Associated with the *KCNMA1*‐N999S Variant

**DOI:** 10.1002/mdc3.13394

**Published:** 2021-12-21

**Authors:** Sotirios Keros, Jennifer Heim, Wejdan Hakami, Efrat Zohar‐Dayan, Bruria Ben‐Zeev, Zach Grinspan, Michael C. Kruer, Andrea L. Meredith

**Affiliations:** ^1^ Division of Neurology, Department of Pediatrics Weill Cornell Medical College New York New York USA; ^2^ KCNMA1 Channelopathy International Advocacy Foundation (KCIAF) New York New York USA; ^3^ Pediatric Movement Disorders Program Barrow Neurological Institute, Phoenix Children's Hospital Phoenix Arizona USA; ^4^ Pediatric Neurology Unit, Edmond & Lily Safra Children's Hospital Chaim Sheba Medical Center Tel Hashomer Israel; ^5^ Sackler Faculty of Medicine Tel Aviv University Tel Aviv Israel; ^6^ Department of Child Health, Neurology, and Cellular Molecular Medicine and Program in Genetics University of Arizona College of Medicine Phoenix Arizona USA; ^7^ Department of Physiology University of Maryland School of Medicine Baltimore Maryland USA

**Keywords:** PNKD type 3, stimulants, movement disorder, KCa1.1, BK channel

## Abstract

**Background:**

*KCNMA1*‐linked channelopathy is a rare movement disorder first reported in 2005. Paroxysmal non‐kinesigenic dyskinesia (PNKD) in *KCNMA1*‐linked channelopathy is the most common symptom in patients harboring the *KCNMA1*‐N999S mutation. PNKD episodes occur up to hundreds of times daily with significant morbidity and limited treatment options, often in the context of epilepsy.

**Cases:**

We report 6 cases with the *KCNMA1*‐N999S variant treated with lisdexamfetamine (0.7–1.25 mg/kg/day), a pro‐drug of dextroamphetamine. Data were collected retrospectively from interviews and chart review. Parent‐reported daily PNKD episode counts were reduced under treatment, ranging from a 10‐fold decrease to complete resolution.

**Conclusion:**

Our findings suggest that lisdexamfetamine is an effective therapy for PNKD3 (*KCNMA1*‐associated PNKD). Treatment produced dramatic reductions in debilitating dyskinesia episodes, without provocation or exacerbation of other *KCNMA1*‐associated symptoms such as seizures.

The *KCNMA1* gene (OMIM 600150) encodes the pore‐forming alpha‐subunit of the voltage‐ and calcium‐ sensitive “BK” potassium channel. BK channels are broadly expressed in the brain and peripheral tissues, such as smooth muscle and neuroendocrine organs, typically suppressing neuronal excitability.^1^ Neurologic abnormalities associated with *KCNMA1* mutations or variants of unknown significance (VUS) are described as *KCNMA1*‐linked channelopathy.^1^ The neurologic phenotype in *KCNMA1*‐linked channelopathy can include seizures, speech/language and motor developmental delays, cerebellar atrophy, microcephaly, hypotonia, facial dysmorphisms, visceral malformations, and movement disorders such as ataxia, dystonia, and paroxysmal non‐kinesigenic dyskinesia (PNKD).^2^ Almost all patients with *KCNMA1*‐linked channelopathy exhibit PNKD, epilepsy, or both. PNKD Type 3 (PNKD3) is defined as PNKD occurring in association with a pathogenic *KCNMA1* variant, with or without epileptic seizures (OMIM 609446).

Currently, the *KCNMA1*‐N999S variant [NM_002247.3 c.2984 A>G (p.N999S)] is the most common mutation reported in the literature, occurring in all known cases as a heterozygous de novo mutation.^2^ Using de‐identified survey data (University of Maryland School of Medicine IRB NHSR Protocols HP‐00086440 and HP‐00092434) and published literature,^2^ we found the majority of patients harboring N999S present with debilitating PNKD (9 out of 12 subjects) and just over half also experience seizures (7 subjects). When introduced into BK channels, the N999S mutation produces strong gain‐of‐function (GOF) channel activity,^3^ supporting the autosomal dominant allele designation. Two other *KCNMA1* GOF mutations are also highly associated with PNKD, providing early phenotypic insights into GOF versus loss‐of‐function variants (LOF)^2^, ^4^ and hypothesizing a common mechanistic basis for PNKD3.

Dyskinesias in PNKD3 typically manifest as protracted sudden onset behavioral arrest or drop attacks, with equivocally dystonic‐ or atonic‐appearing loss of control of most skeletal muscles^2^, ^5^ (see Heim et al.^5^ for video examples). Some rudimentary voluntary muscle control is preserved, but there is difficulty maintaining posture. Many have rhythmic, stereotyped mouth‐gaping movements. Individuals may slump forward or backwards and may fall if standing. Full consciousness is preserved, and patients may answer questions that were asked during the event, after the episode resolves. PNKD episodes tend to be consistent in phenomenology across individuals and can occur dozens of times per day, lasting from a few seconds to several minutes. Although chest wall muscles are affected, the eyes and the diaphragm remain under voluntary control (SK/MCK personal observations and physical examination during PNKD), with few experiencing hypoxia even during prolonged events. Like familial PNKD and other monogenic non‐kinesigenic dyskinesias,^4^ episodes are commonly triggered by excitement or joy, and tactile stimuli such as cold (eg, after bath, stepping into cold air). Due to phenomenological overlap with cataplexy, these events have also been referred to as cataplexy without narcolepsy.^5^

PNKD is often the earliest presenting symptom of *KCNMA1*‐linked channelopathy and typically starts before the age of 24 months. In the authors’ experience, PNKD episodes are frequently mistaken for seizures due to background epileptiform activity on EEG or co‐morbid epileptic seizures, which are also prevalent in *KCNMA1*‐linked channelopathy,^2^ despite the lack of confirmatory abnormalities on scalp EEG during the dyskinesias. Treatment options in PNKD3 are limited. Episodes are refractory to a wide range of anticonvulsants, although acetazolamide can reduce but not eliminate the dyskinesias in some patients.^5^

In 2019, the family of a young adult reported to one of the authors a 10‐year history of daytime remission of dyskinesias after starting lisdexamfetamine, a prodrug of dextroamphetamine.^6^ The episodes were phenotypically similar to those in PNKD3 and subsequent genetic testing revealed a *KCNMA1*‐N999S variant. In addition, a prior case report presented in abstract form at the 2018 American Epilepsy society meeting^7^ described an individual with a different *KCNMA1* variant (N536H, also a GOF mutation) whose PNKDs were successfully treated with dextroamphetamine.^8^ These anecdotal observations were shared with neurologists and families via the patient advocacy group *KCNMA1* Channelopathy International Advocacy Foundation (KCIAF; www.kciaf.org), social media, and news media.^9^ Several affected individuals were subsequently started on lisdexamfetamine by their treating physicians, with consistent, though anecdotal, reports of reduction or remission of PNKD. Here we provide our experience with lisdexamfetamine‐responsive PNKD in the setting of *KCNMA1*‐N999S. Study objectives were to describe the efficacy, dosage range, duration of effect, and side effects.

## Case Series

Data from six cases treated with lisdexamfetamine were collected in 2020–2021. All cases were previously known to the authors as having reported a reduction in PNKDs after initiation of lisdexamfetamine. Data were obtained retrospectively via combination of chart review of medical records and from family interviews (Tables 1 and 2). Specifically, data on dyskinesia duration, daily frequency, and time of onset and duration of medication responses were obtained from interviews and based on estimates and approximations from parental recollection in an open manner. Subjects' dyskinesias started between 7 and 24 months of age. Lisdexamfetamine (0.71 to 1.25 mg/kg daily) led to a reduction of parentally‐observed PNKD in all cases (Table 1). The reported onset of this effect across cases ranged from 20 to 60 minutes after taking an oral dose, with a duration of 8 to 13 hours. This reported time of onset and duration is consistent with the known pharmacokinetic profile of the active metabolite of lisdexamfetamine, dextroamphetamine.^10^ In three cases (Cases A, B, C), there were no observed dyskinesias during this therapeutic time window, down from a baseline of up to 300 daily events. Although most subjects take lisdexamfetamine once daily in the morning, one child (Case D) takes an additional dose of lisdexamfetamine immediately before bed, which led to cessation of previously prolonged, severe nocturnal events.

**TABLE 1:** Genotype, demographics, and lisdexamfetamine effects

Case	A	B	C	D	E	F
*KCNMA1 Genotype* (all heterozygous)	N999S (rs886039469)	N999S (rs886039469)	N999S (rs886039469)	N999S/R1128W^a^ (rs886039469/rs747029218)	N999S/R1128W^a^ (rs886039469/rs747029218)	N999S (rs886039469)
Other known variants (all heterozygous)	RNF31‐Q622LRNF31‐V1036L TNXB‐R38Q TNXB‐G2846L	none	none	none	GRIN2A‐E1256K	none
Age of dyskinesia onset	12 mo	24 mo	7 mo	12 mo	18 mo	11 mo
Estimated pre‐treatment dyskinesias per day	50–200	2 to dozens	50–200	200–300	300+	10–45
Dyskinesia duration (s)	5–30s	5–20s	5–120 s	1–20s	15–30s	30–120 s
Time to onset of medication effect (min)	30–45 m	30 m	30–60 m	60 m	20 m	Not known
Duration of effect (hr)	11 hr	12 hr	13 hr	12 hr	8 hr	Not known
No. of spells per day during lisdexamfetamine effect	0/day	0/day	0/day	5/day	0–3/day	1–3/day
Weight (kg)	28 kg	16 kg	61 kg	20 kg	18 kg	26 kg
Current dose (mg)	20 mg^b^	20 mg^c^	30 mg^b^	15 mg am^b^/5 mg pm^d^	15 mg^b^	30 mg^b^
Dose by weight (mg/kg/day)	0.71	1.25	0.5	1	0.8	1.1
Highest total daily dose tried (mg)	20 mg	20 mg	40 mg	20 mg	15 mg	30 mg
Age lisdexamfetamine started (yr)	8 yr	3 yr	9 yr	5 yr	4.5 yr	7 yr
Current age (yr)	9 yr	4 yr	20 yr	6 yr	5 yr	7 yr

*KCNMA1* variants were identified via commercial diagnostic genetic testing through clinical exome sequencing or genetic epilepsy panels and confirmed as a condition of participation.

^a^ *KCNMA1*‐R1128W is designated benign^2^, ^3^ (and ALM unpublished data).

^b^ Capsule form.

^c^ One 10 mg capsule and one 10 mg chewable tablet.

^d^ Chewable tablet.

**TABLE 2:** Side effects, other medication trials, seizure history, and ancillary findings

Case	A	B	C	D	E	F
Reported negative effects (subjective parental report)	None	Initially “less happy” (resolved)	Mild anorexia and insomnia, both resolved. Persistent personality changes “less laughing, more serious”	Severe mood swings, aggressions	Diminished appetite	Diminished appetite
Non‐PNKD benefits (subjective parental report)	Improved attention and academic performance	Improved speech	None	Improved social skills, improved academic performance, “excelling”		Improved speech, concentration and cognitive function, newly potty trained
Other current medications	Melatonin	Melatonin	None	Docosahexaenoic acid	Levetiracetam, medical cannabis	None
Medication trials with partial effectiveness of PNKD			Dextroamphetamine (one dose), mixed amphetamine salts	Acetazolamide, docosahexaenoic acid	Clobazam, clonazepam	Acetazolamide***
Ineffective medication trials	Acetazolamide, clonazepam, ethosuximide*, imipramine, levetiracetam, oxcarbazepine, zonisamide	Levetiracetam, topiramate, valproate	Acetazolamide, carbamazepine, levetiracetam, phenobarbital	None	Acetazolamide, carbamazepine** docosahexaenoic acid, lacosamide, levetiracetam, medical cannabis, valproate	None
History of Seizures	Yes	Uncertain	Yes	No	Yes	No
Seizure types	Single GTC at 24 mo. Atypical Absence Epilepsy Age 6 yr.	No confirmed seizures	Two lifetime seizures in the setting of febrile illness (age 5 and 6 yr)	None	Isolated mild myoclonic jerks observed only on video EEG	None
Pre‐lisdexamfetamine seizure frequency	100+ per day (EEG confirmed)	None	Two lifetime	None	Very rare, unnoticed by mother observed only during video EEG	None
Post‐lisdexamfetamine seizure observations	Subjective decrease	None reported	None reported	None reported	None reported	None reported
Summary of EEG findings	Age 1–4: Generalized + multifocal discharges. Age 6: Absence or atypical absence seizures. Normal background.	Rare multifocal sharp waves. Occasional generalized polyspikes.	Age 2 yr: Normal. Age 7 yr: Continuous multifocal spikes in sleep. Age 15: Normal	Normal	Normal background. Spike and wave with bilateral central foci. Generalized multi‐spike and wave discharges.	Age 5: diffuse posterior slowing. Multifocal (maximal bi‐occipital) and generalized atypical spike–wave discharges activated with photic stimulation
MRI findings	Normal	Normal	Normal	Normal	Very mild dilatation of ventricles and CSF spaces	Normal

* Worsened absence seizures.

** Worsened PNKD.

*** Diurnal enuresis.

Side effects, including appetite suppression, insomnia, and irritability were reported (Table 2). These adverse effects did not result in discontinuation of lisdexamfetamine but in most cases prevented further increases in dosage. In four of the six cases, parents reported improvement in one or more non‐motor related areas, such as speech, academic performance, concentration, or social skills, based on subjective family observation. PNKD did not resolve with commonly used antiepileptic medications. Two cases had a partial response to acetazolamide, and one case partially responded to a benzodiazepine.

Notably, none of the cases reported new‐onset seizures after starting lisdexamfetamine (Table 2), including those with a prior history of seizures or myoclonic jerks. One case with active daily absence seizures (Case A) reported a subjective decrease in observed absence seizures.

## Discussion

This case series suggests that lisdexamfetamine is well‐tolerated and effective for PNKD3 in children as young as 3 years old. Our observation of the successful use of stimulants in *KCNMA1*‐linked channelopathy corroborates the previously reported case treated with dextroamphetamine.^8^ Although the long‐term effects of using stimulants in PNKD patients is not yet known, stimulants are routinely used for the long‐term management of ADHD in children. Dextroamphetamine is FDA‐approved for ADHD and narcolepsy in children age 3 and older. At present, lisdexamfetamine is approved for the treatment of ADHD in patients 6 and older. While the patients in our series experienced some of the expected side‐effects for stimulant therapy, such as insomnia and anorexia, none were severe enough to discontinue treatment. In addition, no subject had exacerbation of existing conditions, notably there was no worsening or new development of seizures. Due to the severe neurodevelopmental consequences of PNKD3 at the frequency of up to hundreds of episodes per day, the risk to benefit ratio may be favorable for this treatment option. This data provides support for initiation of a clinical trial to further characterize the efficacy of this treatment regime, which would include a control group that was not possible in this retrospective case series study.

While the mechanism remains to be determined, almost all observations of PNKD3 episodes express as hypokinetic movement. Interestingly, attacks of familial PNKD can be triggered by stimulants such as caffeine.^4^ Of note, neither lisdexamfetamine nor dextroamphetamine alter *KCNMA1*‐encoded BK channel activity in heterologous systems^8^ (and ALM unpublished data), suggesting that drug effects on motor control may be mediated indirectly and not via specific modulation of BK channel activity. There is superficial similarity to freezing episodes in Parkinson's disease, in that PNKD3 patients have difficulty initiating lower limb movement such as walking, and which suggests a hypothesis that PNKD3 could be linked to basal ganglia dysfunction.

This case series is retrospective and relies on post hoc parental recollection of pre‐ and post‐treatment PKND counts. However, all the individuals in our series had high‐frequency, persistent symptoms that were consistent with other individuals harboring *KCNMA1*‐N999S variants and other pathogenic *KCNMA1* variants.^2^, ^8^ In all cases, the reported decrease in PNKD events was robust and matched the expected pharmacokinetics for lisdexamfetamine, arguing against reporting bias significant enough to invalidate the key observation. Despite the overall effectiveness of lisdexamfetamine, debilitating dyskinesias often continue to occur in the morning prior to medication onset and in the evenings when effectiveness wanes as blood concentrations decrease. As also seen with stimulants used to treat ADHD, insomnia and appetite suppression essentially prevents around‐the‐clock use of lisdexamfetamine in most cases, and thus alternative treatments are needed.

*KCNMA1*‐linked channelopathy is also associated with mild to severe developmental delay and intellectual disability. Several parents reported improvements in school, speech, or social interactions co‐occurring with reductions in PNKD attacks. This may in part reflect treatment of a co‐morbid deficit in executive function, or may provide evidence that the frequent motor pauses from PNKDs directly interfere with development. However, a more global beneficial effect of lisdexamfetamine on neurological function cannot be excluded.

## Author Roles

(1) Research project: A. Conception, B. Organization, C. Execution; (2) Statistical Analysis: A. Design, B. Execution, C. Review and Critique; (3) Manuscript Preparation: A. Writing of the first draft, B. Review and Critique.

S.K.: 1A, 1B, 1C, 3A, 3B

J.H., W.H., E.Z.D., B.B.Z.: 1C

M.C.K.: 1C, 3B

Z.G.: 1B, 3B

A.L.M.: 1A, 1B, 1C, 3A, 3B

## Disclosures

**Ethical Compliance Statement:** This study was approved by the Institutional Review Board of the Weill Cornell Medical College (protocol 20‐07022352) and Phoenix Children's Hospital (protocol 15‐080) in accordance with all applicable national guidelines and laws. Written consent for participation in this study was obtained from all participants (or legal guardians when appropriate). All authors confirm they have read the Journal's position on issues involved in ethical publication and affirm that this work is consistent with those guidelines.

**Funding Sources and Conflicts of Interest:** The authors declare that there are no conflicts of interest relevant to this work. ALM was supported by grants from the National Heart, Lung, and Blood Institute (R01‐HL102758) and National Institute of General Medical Sciences Training Program in Integrative Membrane Biology (T32‐GM008181), and “Ion Channel Research Fund” (University of Maryland Baltimore account 26,753). For all other authors, no specific funding was received for this work. SK, ALM, and MCK are uncompensated officers of the non‐profit *KCNMA1* Channelopathy International Advocacy Foundation (www.kciaf.org) and write informational articles for the website. KCIAF had no role in the study.

**Financial Disclosures for the Previous 12 Months:** ALM was supported by grants from the National Heart, Lung, and Blood Institute R01‐HL102758 (PI: Meredith) and National Institute of General Medical Sciences Training Program in Integrative Membrane Biology T32‐GM008181 (PI: Meredith), and “Ion Channel Research Fund” (University of Maryland Baltimore account 26,753 (PI: Meredith). MCK was supported by NINDS 1R01 NS106298 (PI: Kruer), Doris Duke Charitable Foundation Data award (PI: Kruer), Cerebral Palsy Alliance Research Foundation PG07217 (PI: Kruer), NICHD 1R01 HD079498 (PI: Duncan), and NIDDK R01 (PI: Zhao).
